# Self-limiting electrospray deposition on polymer templates

**DOI:** 10.1038/s41598-020-74146-1

**Published:** 2020-10-14

**Authors:** Lin Lei, Arielle R. Gamboa, Christianna Kuznetsova, Sunshine Littlecreek, Jingren Wang, Qingze Zou, Jeffrey D. Zahn, Jonathan P. Singer

**Affiliations:** 1 Department of Mechanical and Aerospace Engineering, Rutgers University, Piscataway, NJ 08854 USA; 2 Department of Biomedical Engineering, Rutgers University, Piscataway, NJ 08854 USA

**Keywords:** Polymers, Design, synthesis and processing

## Abstract

Electrospray deposition (ESD) applies a high voltage to liquids flowing through narrow capillaries to produce monodisperse generations of droplets down to hundreds of nanometers in diameter, each carrying a small amount of the delivered solute. This deposition method has been combined with insulated stencil masks for fabricating micropatterns by spraying solutions containing nanoparticles, polymers, or biomaterials. To optimize the fabrication process for micro-coatings, a self-limiting electrospray deposition (SLED) method has recently been developed. Here, we combine SLED with a pre-existing patterned polymer film to study SLED’s fundamental behavior in a bilayer geometry. SLED has been observed when glassy insulating materials are sprayed onto conductive substrates, where a thickness-limited film forms as charge accumulates and repels the arrival of additional charged droplets. In this study, polystyrene (PS), Parylene C, and SU-8 thin films of varying thickness on silicon are utilized as insulated spraying substrates. Polyvinylpyrrolidone (PVP), a thermoplastic polymer is sprayed below its glass transition temperature (T_g_) to investigate the SLED behavior on the pre-deposited insulating films. Furthermore, to examine the effects of in-plane confinement on the spray, a microhole array patterned onto the PS thin film by laser dewetting was sprayed with dyed PVP in the SLED mode. This was then extended to an unmasked electrode array showing that masked SLED and laser dewetting could be used to target microscale regions of conventionally-patterned electronics.

## Introduction

Electrostatic sprays have been widely used in many manufacturing fields, including automotive, pharmaceutical, and agricultural. More recently, electrospray deposition (ESD), one form of electrostatic spraying, has gained attention for micro and nanoscale manufacturing due to its relative ease of control. The mechanism of ESD is to utilize the balance between an electrostatic force and the liquid’s surface tension to produce one or more generations of charged, monodisperse droplets^1^. The spray process begins by diluting materials to be sprayed in a solvent and then pumping the resulting solution through a high voltage nozzle. At the tip of the nozzle, the charged solution forms a “Taylor cone” and breaks into micro-scale droplets at its high-field apex. During flight, solvent continues to evaporate, and the drop size may shrink to reach the “Rayleigh limit,” where surface charges overcome surface tension^1,2^. As a result, the droplet experiences Coulomb fission and forms child droplets that possess larger surface to volume ratios. This process may occur multiple times depending on the spray distance and solution composition, resulting in several generations of monodisperse droplets, most typically two^3^. Compared with traditional deposition methods, ESD offers numerous advantages, including: (1) generation of monodisperse droplets and uniform deposition; (2) the micro/nano size of particles produced by spray processing makes ESD an effective method for micro/nanoscale coatings; (3) morphologies of thin films are easy to adjust by varying flow rate, applied voltage, and spray temperature; (4) the process only uses small quantities of the precursor solutions and spray material; (5) and, the spray process can be implemented in ambient environment. This has led to the application of ESD for the deposition of a wide variety of nanomaterials including cells^4–7^, nanoparticles^8–11^, and polymers^3,12–14^. Many of these examples include the use of ESD to incorporate active materials into electronic devices^3,8,9,11,12,14^. For example, Varga et al.^11^ fabricated three-dimensional Pt/CsH_2_PO_4_ composites through ESD, which led to a nanostructured electrode layer with high porosity that enhanced electrochemical activity in a fuel cell system. As another example, Deng et al.^14^ demonstrated that ESD could deposit semiconducting polymers with enhanced crystallinity and domain orientation.

Because of the charged nature of the sprayed droplets, the target of the spray is critically important to the results; however, a majority of the studies to date have focused on the droplets in the air. The effects of target geometry and topography have shown great potential for high-efficiency patterning. For example, charged and insulating stencil masks have been applied to template the spray^15–20^. Higuchi et al.^15^ demonstrated the focusing effects of different designs of non-conductive stencil masks to change the size of nanoparticle deposits. Osuji et al.^19^ demonstrated that inverse masks of a grounded grid under a glass slide could lead to patterned deposits of polymer films. More recently, Zhu and Chiarot^20^ demonstrated that the charging effects of ESD with near-field photoresist templates could result in focusing of towers of particles that greatly exceed the thickness of the mask. As shown in these examples, the focusing effects of ESD make it much more difficult to predict the amount of material that will deposit on the unmasked regions as compared to more traditional, linear patterning methods such as liftoff. However, the powerful capabilities of templating using charge effects were also demonstrated, which would greatly expand the variety of materials that could be employed with templated lithography and simultaneously reduce materials waste. Hence, in this work, we explore the effects of the template on the SLED process as a means of control.

Recently, we have demonstrated self-limiting electrospray deposition (SLED) as a means to create microcoatings on complex 3-dimensional (3D) surfaces^18,21^. When an insulating glassy material in a volatile solvent is sprayed below its glass transition temperature (T_g_) onto a conductive surface, charges accumulate on the deposited porous, insulating thin film. As a result of electrostatic repulsion from these accumulated charges, the spray achieves a thickness-limited coating. When combined with near-field templating, the SLED effect can allow for both specified positioning and quantity of the sprayed materials. In particular, we see an opportunity to combine localized laser dewetting and SLED.

Focused laser spike (FLaSk) dewetting uses laser heating in a thin film to create thermocapillary forces with a high degree of spatial and temporal control. Through local softening, samples that are solid both before and after excitation lock in the effects of the exposure, such as dewetting^22–25^ and/or zone annealing^26–28^. We have also shown that FLaSk has the ability to selectively pattern multilayers of material^29^. For the purposes of templating, FLaSk is developer-free and grayscale allowing for partial to complete dewetting in submicron feature sizes followed by immediate spray. The size of the dewetted pattern has been determined to be dependent only on laser power and numerical aperture (NA) and the material's molecular weight^24,29^. FLaSk is also compatible with low T_g_ materials, meaning that the templating process can be performed at low temperatures. This presents advantages over current micro-processing techniques in electronics. By eliminating the need for liftoff or masking and etching procedures, we can mitigate the risk of damaging underlying layers with corrosive chemicals or leaving unwanted residue on patterned components. Furthermore, FLaSk-dewetted stencils may be removed post-ESD by selecting an orthogonal system to the sprayed polymer. Another advantage is that if the optical absorption of the target electrode is used as the heat source, the dewetting will be confined to the target, allowing for high-resolution electrodes to restrict the extent of demasking. This is similar to the Joule heating thermocapillary approach employed by Rogers et al.^30^ to demask and etch single carbon nanotubes.

To begin, we aim to understand the effects of a pre-existing polymer layer on the SLED coating thickness. This allows us to determine the minimum mask thickness required to template SLED patterns and additionally provides a route to bilayer coatings created by SLED. Next, we consider FLaSk-patterned templates. In contrast to the work of Zhu and Chiarot, our study aims to determine the templating limits of the masking film when spraying insulating polymers. Because charge cannot easily travel in these polymer films, the resulting spray pattern is of interest. In particular, the dimensions of the mask, when controlled down to the order of microns, can have interesting implications on the self-limiting thickness of sprayed films.

As model polymer pre-layers, we explored polystyrene (PS) and SU-8 photoresist deposited through spin coating and poly(para-xylylene) (Parylene C) deposited through chemical vapor deposition (CVD), all on silicon (Si) wafers. PS is a commodity polymer we have repeatedly used for both SLED and FLaSk experiments for its glassy properties, while parylene is a ubiquitous polymer used for highly controlled conformal coatings. SU-8 is suitable for FLaSk experiments because of its low T_g_ (~ 50 °C) and ease of processing. We applied sprays of poly(vinylpyrrolidone) (PVP), which was selected for its high T_g_ (~ 170 °C) onto these films. To further examine the effects of in-plane templating, microhole arrays with different diameters formed in a PS thin film were made by FLaSk dewetting and sprayed with PVP. To demonstrate the combined SLED-FLaSk technique on microelectronics, we also used SU-8 to mask titanium-platinum (Ti/Pt) electrodes. These films were laser dewetted using an overlapping line writing technique to clear larger regions for PVP spray.

## Results

**Figure 1:**
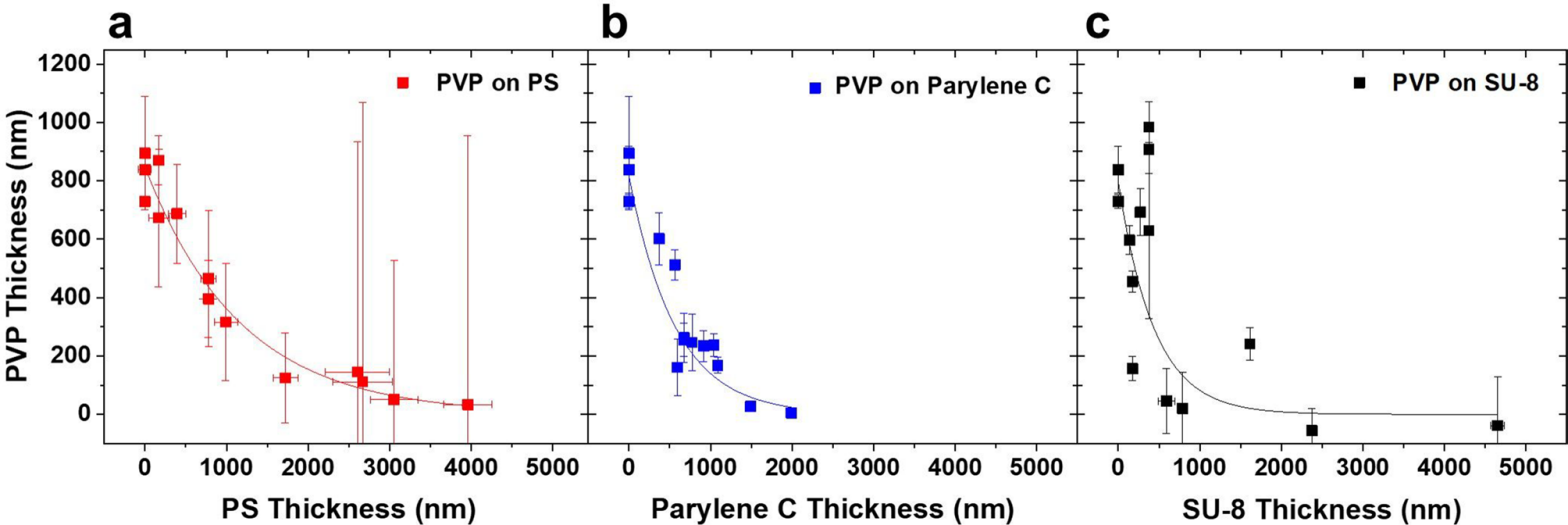
(**a**) 1 wt% PVP sprayed at 0.1 mL/h for 60 min in the humidity and temperature control chamber at 27 °C with different thicknesses of PS-on-Si substrates. At the larger PS thicknesses, the amount of PVP deposited is very thin, so it becomes difficult to distinguish from the roughness of the PS sample leading to large deviations in the apparent measured thickness. The red trace shows an exponential decay fit of *y* = 846.9 nm*e^(−*x*/1174.8 nm)^, R^2^ = 0.96. (**b**) 1 wt% PVP sprayed at 0.1 mL/h for 60 min on Parylene C-on-Si substrates at varying thickness. Due to the conformal nature of vapor deposition, the horizontal error bars are not visible. The blue trace is an exponential decay fit of *y* = 815.6 nm*e^(−*x*/567.4 nm)^, R^2^ = 0.98. (**c**) 1 wt% PVP sprayed at 0.1 mL/h for 60 min on SU-8-on-Si substrates with different thicknesses. The black trace is an exponential decay fit of *y* = 796.0 nm*e^(−*x*/440.6 nm)^, R^2^ = 0.60. Plots generated by the authors using OriginPro 2018 (https://www.originlab.com/).

To investigate the self-limiting effects on different polymers, thin films consisting of PS, Parylene C, and SU-8 were utilized as insulating masks with different thicknesses and sprayed with PVP by SLED. Figure 1 shows the sprayed PVP thin film’s thickness change with the polymer mask’s thickness. Figure 1a shows the thickness change of PVP films with different PS-coated Si substrates. Without the insulating film, for a 60 min spray, PVP reaches a thickness of ~ 800 nm. As the PS coating thickens, the sprayed thickness of PVP films gradually decreases until it is negligible at a PS thickness of ~ 2000 nm. Figure 1b shows the thickness of PVP films on Parylene C. The PVP also decreases with increasing thickness of Parylene C but at a more rapid rate, becoming negligible at a parylene thickness of ~ 1500 nm. Figure 1c shows the thickness of PVP films with different SU-8 thicknesses spin-coated onto Si substrates. Sprayed PVP thickness decreases with increasing SU-8 thickness. Deposited PVP here decays at a more rapid rate than on both PS and Parylene C, becoming negligible at parylene or SU-8 film thicknesses of ~ 1000 nm, however there is greater variability in the SU-8-masked samples.

**Figure 2:**
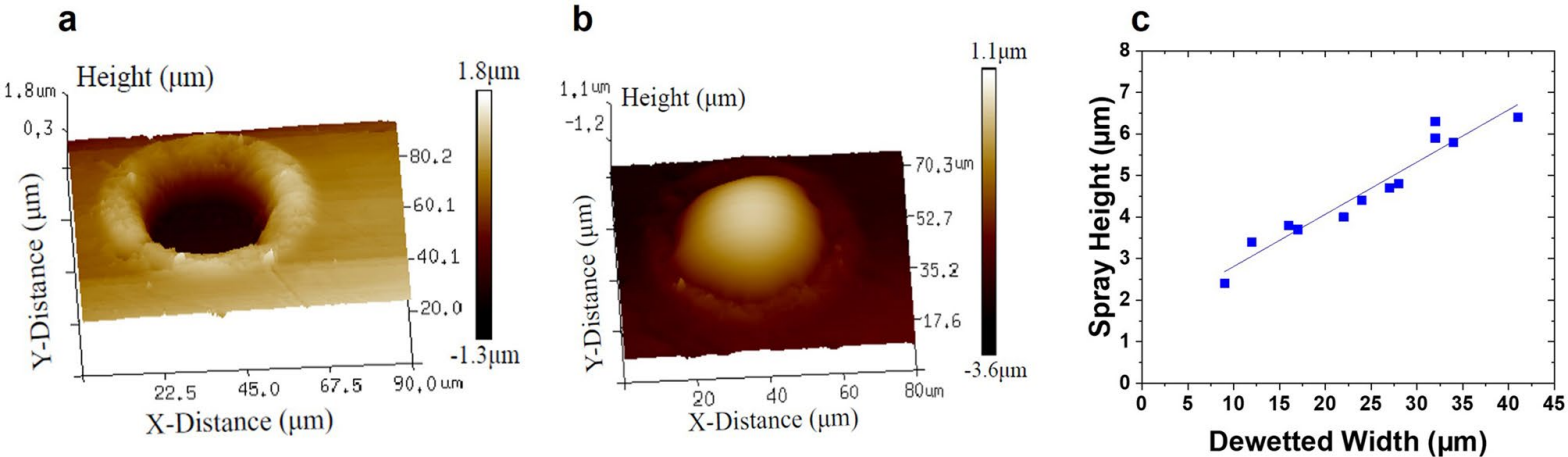
3D maps of a 90 mW, 0.25 NA laser-dewetted PS feature before (**a**) and after (**b**) spray with PVP. Maps generated by the authors using Bruker NanoScope 9.1 (https://www.bruker.com/). (**c**) The height of the sprayed PVP feature after smoothing in ethanol vapor determined from AFM profiles. The linear fit has an equation y = 0.13x + 1.55 μm, R^2^ = 0.93. Plot generated by the authors using OriginPro 2018 (https://www.originlab.com/).

Figure 2 shows Atomic Force Microscopy (AFM) results of the FLaSk dewetted features. 3D profiles, with characteristic examples shown in Fig. 2a,b were used to extract the width of the fully dewetted region (Fig. 2c) as well as height of the PVP feature (Fig. 2c). The linear fit in Fig. 2c predicts a minimum spray quantity (*y*-intercept at 1.55 μm) that is deposited even when the mask is not dewetted.

**Figure 3:**
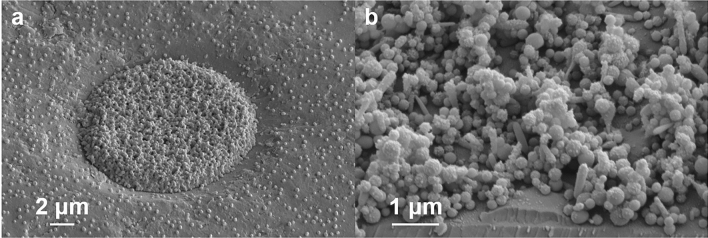
(**a**) Tilted scanning electron microscope (SEM) image of a FLaSk-dewetted feature on PS created with 50 mW, 0.25 NA laser and sprayed with PVP. (**b**) Tilted SEM image of 0.25 wt% 10 K PVP in 80 vol% ethanol to 20 vol% water sprayed at 0.5 mL/h for 20 min.

To investigate the structure of the sprays before smoothing, we employed tilted scanning electron microscopy (SEM). Figure 3a shows a characteristic result of a spray-filled FLaSk dewetted feature patterned on PS at 50 mW. Other morphologies that were observed looked similar, and it was difficult to extract additional quantifiable data from these images. These images did reveal the presence of some single-particle monodisperse residue, though these would have been difficult to detect optically without a sizable index mismatch. As a point of comparison, Fig. 3b shows the results of an untemplated PVP spray to contrast with the focusing effect of the template.

**Figure 4:**
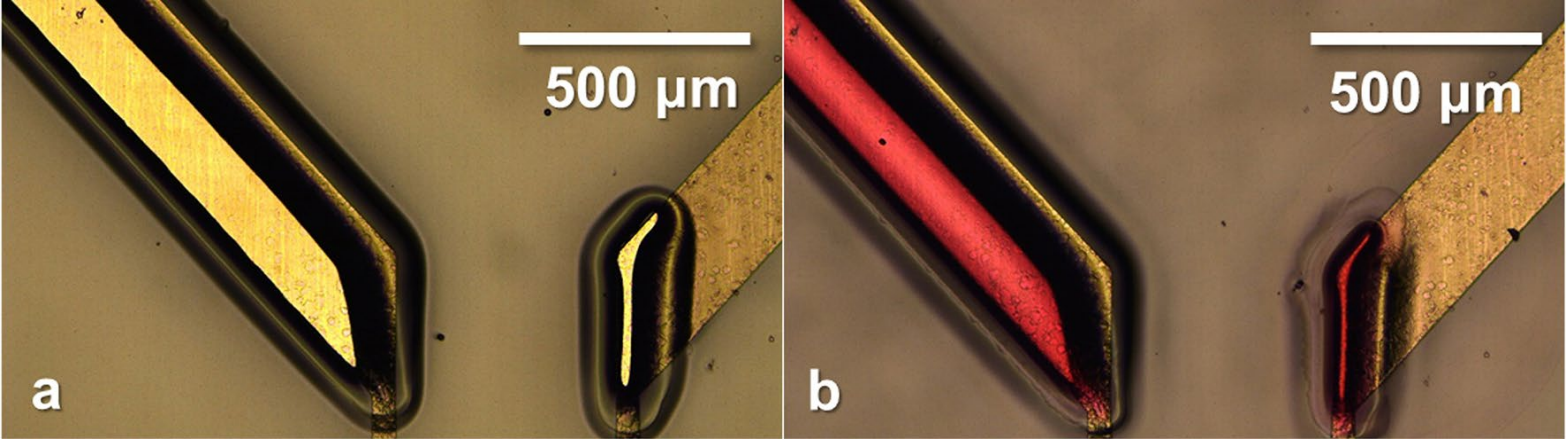
Optical images of 100 mW laser-dewetted features on SU-8 on Ti/Pt electrodes before (**a**) and after (**b**) dyed PVP spray, with the right pad grounded using copper tape.

SU-8-masked Ti/Pt electrodes were also sprayed with dyed PVP to demonstrate the ability of the polymer to insulate and direct incoming spray. Figure 4 shows optical images of the electrode after FLaSk dewetting (Fig. 4a) and after spraying and smoothing of the deposited PVP (Fig. 4b). Dewetting allowed for the selective patterning of the SU-8 film on the electrode, leaving other regions of the film insulated from the incoming spray. PVP was successfully deposited onto the exposed platinum, and an optical examination of the surrounding film showed no significant spray on masked areas. Interestingly, while only the right electrode pad was grounded, both templates attracted spray. This suggests a secondary charge transport mechanism that prevented charge accumulation and consequently allowed for spray deposition.

**Figure 5:**
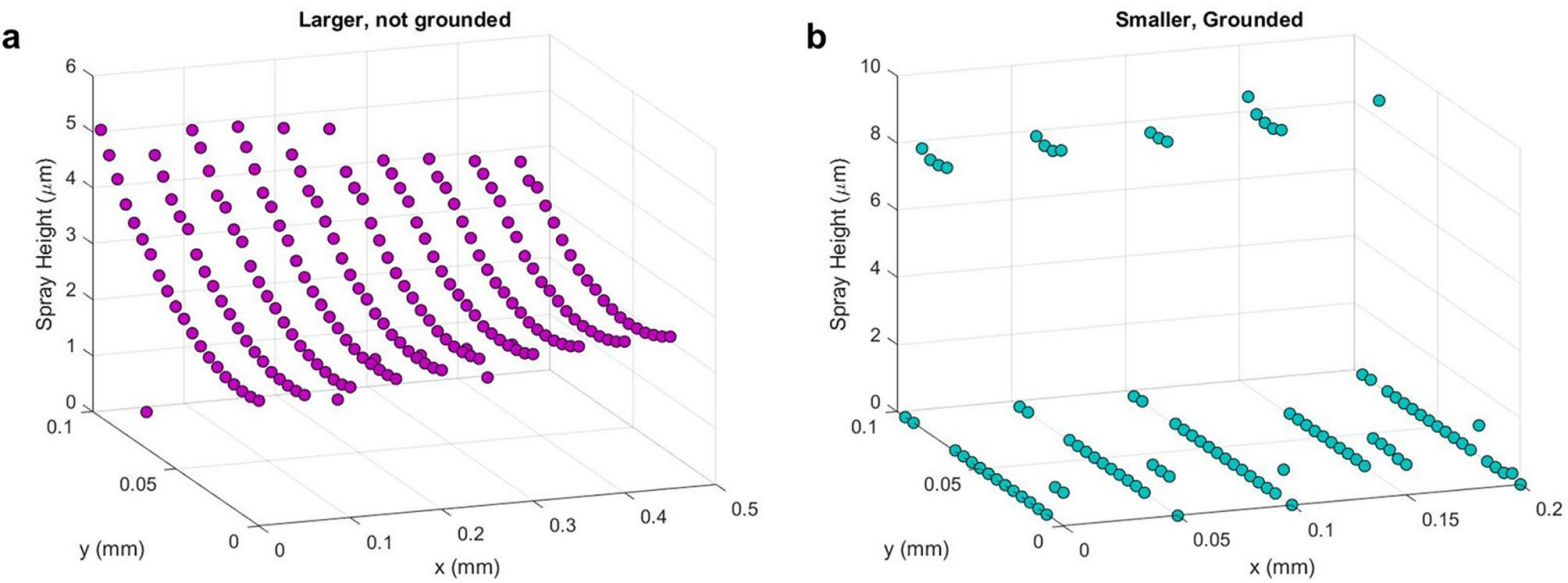
Average thickness of sprayed PVP film on FLaSk-dewetted SU-8 on Ti/Pt electrodes as seen in Fig. 4. Plots generated by the authors using OriginPro 2018 (https://www.originlab.com/).

Figure 5 shows the thicknesses of sprayed PVP in Fig. 4 averaged along a 100 μm cross-section. In the electrode with a narrower dewetted feature (Fig. 5b), sprayed PVP has attained its peak thickness at ~ 8 μm, and its surroundings have collected no spray. The wider mask in Fig. 5a, however, has a maximum thickness at ~ 5 μm which gradually decreases along its width. Here, templated PVP has not attained its SLED thickness, further illustrating the effects of in-plane confinement first seen in Fig. 2c.

## Discussion

While the polymer thickness increases, the ability to dissipate charges through the thickness of the film decreases, resulting in thinner sprayed PVP films. Interestingly, the thickness at which the PVP coating becomes negligible on PS is very similar to the 2000–3000 nm coating thickness that was observed to be the SLED coating for PS sprays onto Si after densification^18^. This supports our prior conclusion that the SLED thickness is less a dynamic process than is inherent to the conductivity of the polymer and that the other observed changes in thickness have more to do with thermal and solvent-related effects.

For Parylene C deposition, the thickness decrease of PVP thin films occurs more rapidly. These results are not surprising because the bulk resistivity *ρ* and dielectric constant *k* of PS, *ρ* =  ~ 10^15^ Ohms-cm and *k* = 2.6 respectively^31,32^ (Table S2), are less than those of Parylene C, *ρ* =  ~ 10^16^ Ohms-cm and *k* = 2.95–3.15^33^. Sprayed PVP thickness decreases at an even greater rate on SU-8 films (*ρ* =  ~ 10^14^ Ohms-cm, *k* = 3.28^34^) than on both Parylene C and PS (Fig. 1c), suggesting that the dielectric constant is the critical parameter. SU-8 samples showed uncharacteristically thick top layers, particularly on thinner samples, and a greater overall sample to sample variation. This is most likely due to the ability for SU-8 to be swollen by the spray solvent (ethanol). During flight, solvent is expelled from spray droplets in a series of Coulomb fissions, ultimately leading to dry spray. The ionized solvent continues to follow spray and forms solvent vapor around the substrate, allowing charges to conduct^18^. As a result, a thicker film must be used to compensate for this added charge dissipation mechanism.

These results confirm that the barrier thickness occurs once a specific total film thickness is reached for a given polymer. The presence of a pre-deposited thin film can adjust the thickness of the coating sprayed by SLED, and the selection of polymer coating with controlling thicknesses can be used as an effective means to control the thickness of a second spray, though at very thin coatings, solvent swelling effects can lead to enhanced accumulation on the mask. When using a mask-swelling solvent, such as the FLaSk-dewetted templates shown above, additional thickness is needed as the focusing of the spray also focuses the vapor, which can lead to localized overfilling (Fig. S2).

To show the effects of in-plane confinement, we employed the FLaSk dewetting as a templating method for fabricating micropatterns through near-field stencil templating. While the sprayed PVP on dewetted PS thin films was clearly much thicker in the dewetted regions than on the mask, the effects of the in-plane confinement were also apparent in the variation in height. Two important things to note are that the thickness within the dewetted regions is much higher than the PVP self-limiting thickness and also that overall curvature, even in the smallest dewetted feature is quite small. This indicates that the charge build-up from the surrounding mask is at first focusing the spray to create taller features before creating a repulsive charge that arrests the spray, leading to the observed increasing height with increasing dewetted width.

Charge accumulation effects are also evident in the SU-8 masked platinum electrodes: the different widths of the dewetted features resulted in different spray heights and curvatures. The wider mask on the left of Fig. 3 produced a PVP film with a lower peak thickness that gradually decreased as it approached the edge of the mask. This morphology is consistent with previous results (Fig. 2c): as the stencil accumulates charge and repels incoming spray, it causes a thickening effect in the center of the exposed conducting region. This focusing effect is amplified in the narrower dewetted mask: as droplets approach the insulating polymer, charge cannot adequately dissipate, repelling PVP spray and directing it into the center of the platinum. Rather than spread laterally, the sprayed PVP film grows in thickness, and the surrounding area attracts virtually no spray. The resulting ESD thickness in dewetted trenches is also influenced by the thickness of surrounding SU-8 ridges. FLaSk dewetting was carried out in one direction, causing SU-8 to gather on one side of the dewetted feature. Consequently, more charge accumulates where SU-8 ridges are thicker, further repelling incoming PVP spray and causing the asymmetric curvatures seen in Fig. 5.

When contrasted with the film growth pattern in Fig. 2c, a relationship is seen between film thickness and dewetted pattern width. PVP film thickness varies across the width of the wider dewetted pattern in Fig. 4a, Fig. 5a showing a maximum thickness at ~ 5 μm and decreasing to ~ 2 μm. This film thickness evolution agrees with the trend observed in Fig. 2c in both these cases, while not shown, the deposited film thickness will increase at a decreasing rate to eventually attain a new self-limiting templated thickness. The apparent growth here suggests that more PVP can still be sprayed to observe SLED behavior. In the thinner pattern of Figs. 4b and 5b, the PVP film differs greatly along the dewetted width. Deposited material is concentrated in the center and attains a peak thickness ~ 8 μm while there is nearly no material near the edges; this suggests that PVP has reached its self-limiting thickness for this mask width. This difference especially highlights the effect of stencil dimensions on the SLED thickness of polymers. According to charged mass transport mechanisms, we expected that only the grounded electrode would sufficiently dissipate charge and attract PVP spray; however, PVP was deposited on both regions (Fig. 4b), suggesting that charge traveled through the glass substrate that housed the Ti/Pt electrode. Chiarot et al. and Osuji et al. previously demonstrated this ability to spray on glass substrates despite low electrical conductivity^12,35^. We suspect this is due to the surface conductivity of many low-melt glasses being great enough to conduct the low charge deposition rate of ESD. While the specific mechanism of this conduction is beyond the scope of this work, the absorption of water by glass is known to increase its surface conductivity^36^. This said, these experiments were specifically conducted at low ambient humidity and the glass layer was buried under the polymer mask, so any water-mediated conduction would have had to occur due to a water layer trapped during spin coating.

Interestingly, the un-densified regions may have an aspect ratio reaching or exceeding 1, especially if we could capture them in their fully undensified form. The SEM analysis revealed that the as-sprayed structures were considerably more collapsed than untemplated sprays. We have previously observed this behavior when samples approach the solid’s T_g_^18^. PVP is well below its T_g_ at room temperature, but the focusing effect of the template undoubtably led to a much higher effective flow rate as all of the droplets incident on the sample were directed to the laser-dewetted region. This in turn would have created a higher vapor pressure of ethanol surrounding the templates leading to solvent vapor annealing of the structure. This suggests that future experiments should explore the effects of the macroscopic flow rate on the height and porosity of the unsmoothed and smoothed samples. It is anticipated that higher aspect ratios may be accessible as the effective flow rate approaches the macroscopic flow rates generally employed.

## Conclusion

We have shown that the presence of a pre-deposited thin film can adjust the coating thickness of self-limiting electrospray deposition (SLED) and further, that the selection of polymer coating determines the magnitude of this effect. In the case of polystyrene (PS), this effect mirrors the thickness observed during SLED sprays of PS solutions. We also examined FLaSk dewetting as a templating method for fabricating micropatterns through near-field stencil templating. In these templated structures, effects of charge lensing are also apparent, introducing the possibility for templating structures that do not mimic the overall morphology of their templates. For instance, structures with overhangs or raised regions can be produced by combining thickness and in-plane templating with grayscale mask patterning by FLaSk dewetting or embossing. We have demonstrated that this technique when combined with ESD can be especially useful for fabricating micro-electronics because of the non-destructive method by which FLaSk dewetting directly heats the polymer mask through the platinum electrode. Furthermore, since SLED can be applied for coating 3D targets, this could be a potential way to accomplish multilayer coatings or templating on more complicated 3D structures.

## Methods

### Materials

PS (35 kDa), PVP (10 kDa), Oil red EGN, 2-butanone (> 99%), and propylene glycol monomethyl ether acetate (PGMEA, ≥ 99.5%) were purchased from Sigma-Aldrich and were used as received. For parylene coatings, Parylene C dimer (Specialty Coating System, USA) and trichlorosilane (Aldrich Chemistry) were used as received. Sprays were conducted from pure ethanol (KOPTEC, 200 proof pure ethanol). SU-8 resin (EPON) was purchased and used as received for electrode masks. Ti/Pt electrodes were prepared on glass substrates via photolithography and acetone lift-off. They consist of two identical square pads that each branch into 225 μm and then 50 μm wide segments. Each segment is formed parallel to the other such that the 50 μm segment pair acts as a capacitor. The metal layers were deposited such that the platinum interfaces with the SU-8 mask layer and the titanium acts as an adhesion layer in contact with the glass substrate.

### Electrospray deposition set up

**Figure 6:**
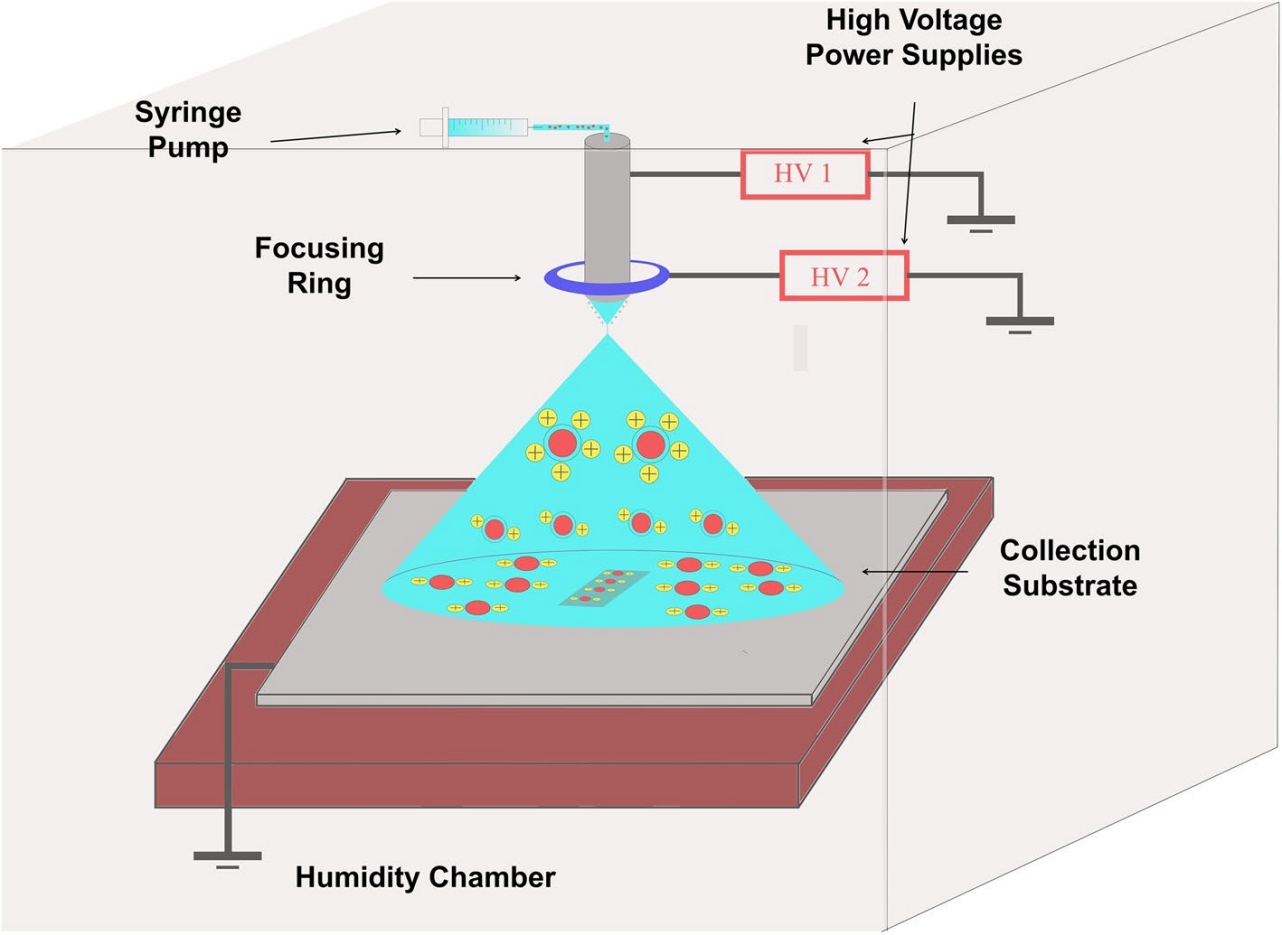
Schematic diagram of electrospray deposition setup. Schematic drawn by the authors using Inkscape 0.92.4 (https://inkscape.org/).

Figure 6 shows the diagram of our electrospray deposition system. This electrospray set up includes six components: (1) syringe pump (Harvard, 70-2208), (2) two high voltage power supplies (Acopian, P012HA5M) (3) stainless steel needle (SAI Infusion, 20 gauge, 1.5”), (4) steel focusing ring (inner diameter is 2 cm, outer diameter is 4 cm), (5) Si collection substrate, (6) humidity chamber with humidity and temperature control systems (ETS). The ESD system was set up in the controllable humidity chamber where internal humidity was set to 10–20% and internal temperature was maintained at 27 °C. Spray solution was loaded into a disposable syringe (NORM-JECT, 6 mL) and delivered by syringe pump. As liquid passed through the stainless-steel needle, the power supply provided an adjustable high voltage, and the charged drops were deposited onto the collection substrate using the steel ring’s focusing effect. A 10 cm circular Si wafer (University Wafer, Inc., N/Ph) was clipped to a grounded wire placed underneath the polymer chip to prevent charge build-up in the surrounding area. All Si wafers were cleaned by ethanol and acetone and reused after each spray.

### Mask preparation

We used spin coating technique (Laurell Technologies WS-650) to produce flat PS and SU-8 films on 2 cm × 3 cm Si chips as insulating masks. PS was diluted in 2-butanone as a precursor solution for spin coating, with the cast thickness controlled by varying the concentration of the precursor solutions (0.5 wt%, 1 wt%, 2 wt%, 5 wt%, 10 wt% and 15 wt%). Each concentration was spun from 1000 to 6000 RPM to obtain smooth films that were 0–4 μm thick. To eliminate residue solvent following spinning, each film was baked on a hot plate at 100 °C for 10 min. The thin films were measured by a Filmetrics F-40EX reflectometer system with a custom XY-mapping stage.

Parylene C thin films were deposited by vapor deposition in a SCS Labcoter 2. Chips were treated with an adhesion promoter, trichlorosilane (Aldrich Chemistry) prior to Parylene C deposition. The treatment was performed using vacuum evaporation of 20 µL of the promoter around the edge of a petri dish which contained the substrates to be coated. The substrates were held under vacuum for 20 min then transferred to the parylene deposition chamber carousel. Parylene C dimer was weighed out in grams where the deposited film thickness is directly proportional to the dimer mass used. The measured Parylene C dimer (di-para-xylylene) was placed in an aluminum foil container which was then placed in the furnace chamber. The deposition chamber and furnace were vacuumed down to below 15 mTorr before enabling the pyrolization process. The Parylene C dimer was pyrolized at 690 °C into monomers of para-xylylene. These monomers entered the chiller-cooled deposition chamber as a vapor and spontaneously repolymerized as a conformal film. This film uniformly deposited on all exposed surfaces in the chamber. The time for each deposition was based on the weight of Parylene C in the furnace. The thicknesses of Parylene C films were between 370 and 1990 nm.

The microhole array on PS thin films was prepared by FLaSk dewetting as described previously^29^. Briefly, we sputtered a 35 nm gold film onto a 1-mm thick glass substrate using an Anatech Ltd Hummer X gold sputter. We then spun a 1400 nm PS film, which is the thickest PS film that can be easily dewetted, using a 20 wt% solution in PGMEA and post-baked for 10 min at 70 °C for smoothing and removal of residual solvent. Laser dewetting was carried out using a 532 nm continuous wave light source from a Laser Quantum Opus 6 W diode laser controlled by a MATLAB program and custom optical setup (Fig. S1). The laser was shuttered by an Isomet IMAD-T110L-1.5 acousto-optic modulator, circularly polarized and passed through a series of optics, including a final 0.25 NA objective lens to focus the spot onto the gold heating layer. A green dielectric mirror was placed before the objective to feed into a camera to allow imaging during experiments with a red light source placed above the sample. A partially reflecting mirror was placed in the beam path before the objective lens to reflect light into a Thorlabs S121C power meter which read the laser’s power output. The samples rested on a Mad City Labs MCL-MOTNZ stage with a 1″ × 1″ lateral movement fitted with a piezo-controlled axial stage with 200 nm range. This stage allowed for translation in 3 axes when FLaSk dewetting. The dot array was dewetted by pulsing the laser at 1-s intervals with powers ranging from 40 to 150 mW.

The Ti/Pt electrodes were grounded on a single pad using copper tape and masked using a ~ 12 μm thick SU-8 film. SU-8 was chosen as the mask in this test because its low T_g_ allows for low power, low temperature patterning via FLaSk dewetting. These masks were prepared by spin coating a 50 wt% SU-8 solution in 2-butanone at 6000 rpm, 1000 rpm/s for 60 s. The film was then soft baked at 75 °C for 5 min to smooth and remove residual solvent. FLaSk dewetting was performed in a series of overlapping lines using a 0.25 NA lens at 100 mW and 500 μm/s write speed, with a 2-μm spacing between consecutive lines. This combination of laser parameters ensured that material was effectively removed from the desired regions without damaging the underlying platinum.

### Experimental parameters and analysis

PVP was dissolved in ethanol as a 1 wt% solution for spraying on PS and Parylene C films. For FLaSk micropattern spray, 0.05 wt% red dye was added to 1 wt% PVP solution to help visually locate the patterns. The flow rate from the syringe pump was 0.1 mL/h, and the spray distance from the tip of the spray needle to the substrate was 4 cm. For all sprays in this study, the voltage we used were maintained at 5.4 kV. The focus ring was placed 1 cm above the needle and was held at a voltage between 2.3 and 2.5 kV to ensure a stable spray. During the spraying procedure, the chamber humidity was maintained between 10 and 20% by the ETS chamber, ensuring stability and preventing humidity-driven charge dissipation. After spraying for 60 min, each sample was smoothed in an ethanol vapour bath under ambient conditions for 30 s. In order to measure the thickness of the sprayed thin films, we mapped the film thicknesses on the chips with the reflectometer before and after spraying, assuming that all coatings had approximately the same optical properties and index of refraction (that of PS, 1.55–1.59). The mapping profile of the samples covered an area of 0.5 cm × 0.5 cm with 25 locations analyzed for each sample in a 5 × 5 grid. The average thickness for a single chip was used to plot the thicknesses for analysis, with the difference of averages before and following spraying used as the apparent PVP thickness. To evaluate the size of the FLaSk features before and after spraying, a Dimension ICON AFM was employed in tapping mode with an 18 kHz Si tip. SEM was employed to characterize the surface properties of the FLaSk features after spray.

## Supplementary information

Supplementary Information.
