# Supplementary material for: Self-limiting electrospray deposition on polymer templates
Source: Sci Rep. 2020 Oct 14;10:17290. doi: 10.1038/s41598-020-74146-1 (PMC7560848; doi:10.1038/s41598-020-74146-1)
Supplement: Supplementary file 1 — Supplementary Information. [file 41598_2020_74146_MOESM1_ESM.pdf]

Supporting Information

Self-Limiting Electrospray Deposition on Polymer Masks

Lin Lei[1], Arielle R. Gamboa[1], Christianna Kuznetsova[1], Sunshine Littlecreek[2], Jingren Wang[1], Qingze Zou[1], Jeffrey D. Zahn[2], Jonathan P. Singer[1,*]

[1] Department of Mechanical and Aerospace Engineering, Rutgers University, New Jersey 08854
[2] Department of Biomedical Engineering, Rutgers University, New Jersey 08854

[*]jonathan.singer@rutgers.edu

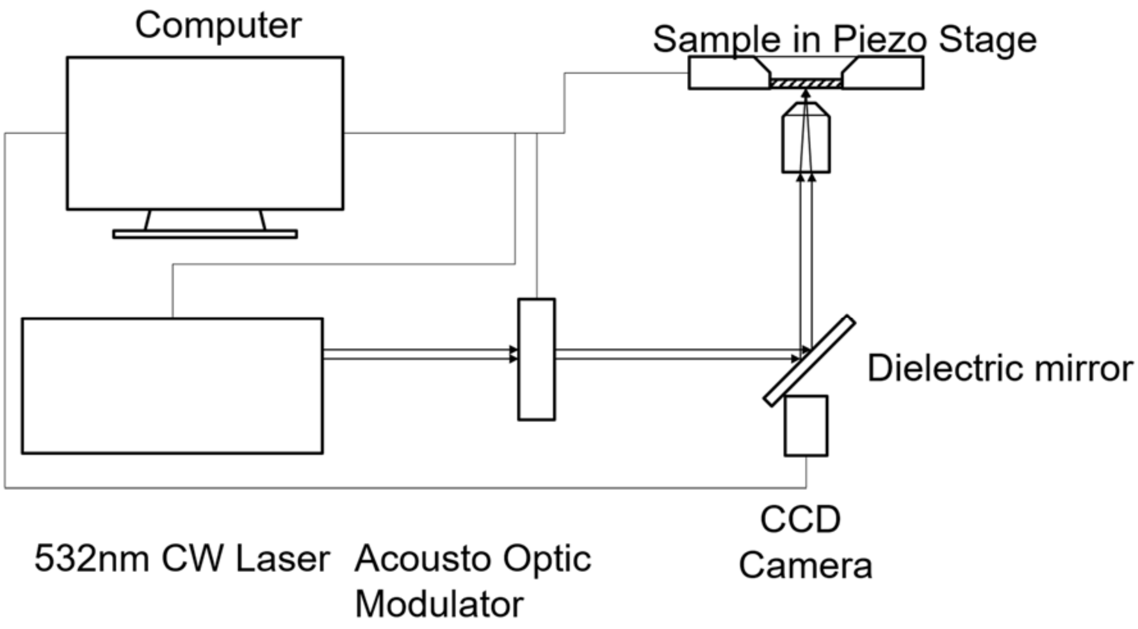

Figure S1. Schematic diagram of laser setup.

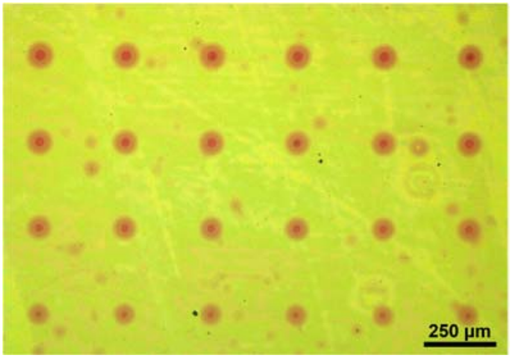

Figure S2. Overfilled holes that occur when the template layer is too thin due to local accumulation of solvent vapor.

**Material**	y_0	x_0
Polystyrene	846.9±29.0	1174.8±122.8
Parylene C	815.6±26.8	567.4±55.9
SU-8	796.0±50.7	440.6±179.8

Table S1. Confidence ranges of exponential fits.

Material	Bulk Resistivity (Ohms-cm)	Dielectric Constant
Polystyrene	~ 10^{15}	2.6
Parylene C	8.8 x 10^{16}	2.95-3.15
SU-8	7.8 x 10^{14}	3.28

Table S2. Electrical properties of candidate insulating polymer films.
